# Endometriosis Presenting as Recurrent Haemorrhagic Ascites: A Case Report and Literature Review

**DOI:** 10.22074/ijfs.2020.5895

**Published:** 2020-02-25

**Authors:** Alejandro Gonzalez, Santiago Artazcoz, Francisco Elorriaga, Douglas Timmons, Jose Carugno

**Affiliations:** 1 Obstetrics and Gynaecology Department, Hospital Naval Pedro Mallo, Buenos Aires, Argentina; 2 Department of Obstetrics, Gynecology, and Reproductive Sciences, Division of Minimally Invasive Gynecology, University of Miami Miller School of Medicine, Miami, FL, USA

**Keywords:** Endometriosis, Haemorrhagic Ascites, Pelvic Pain

## Abstract

Endometriosis is a common condition that occurs in 6-10% of all reproductive age women. This number increases to approximately 40% in women with infertility and nearly 75% in women with complaints of chronic pelvic pain. Endometriosis is characterized by the presence of endometrial glands and stroma outside the uterine cavity. The most common complaints associated with endometriosis are dysmenorrhea and pelvic pain; however, patients often present with a variety of symptoms and on occasion are asymptomatic. When presenting with haemorrhagic ascites, endometriosis mimics ovarian malignancy. Conservative medical treatment is a feasible management option, especially in young patients who desire to preserve fertility. This article aims to present an extremely rare presentation of endometriosis, haemorrhagic ascites, and a review of the associated literature.

## Introduction

Endometriosis is characterized by endometrial glands and stroma outside the uterine cavity. Endometriosis is a common condition that occurs in 6-10% of all reproductive age women (1-3). This number increases to approximately 40% in women with infertility and nearly 75% in women with complaints of chronic pelvic pain (4, 5). The pathogenesis of endometriosis is still debated. A well-founded theory postulates that it could be caused by retrograde menstruation of hormone-sensitive endometrial cells and tissues, which implant on peritoneal surfaces and cause an inflammatory response (6). The most common complaints associated with endometriosis are dysmenorrhea and pelvic pain; however, patients often present without pain and only with complaints of infertility, or there is an incidental finding of an ovarian mass on imaging (7). One exceedingly rare, and interesting, presentation is haemorrhagic ascites. Since its first description in 1954 by Dr. Brews, less than 100 cases of haemorrhagic ascites associated with endometriosis have been documented (8).

This article aims to present a case of a 32 year-old woman who presented with recurrent haemorrhagic ascites. We will discuss the patient’s clinical course and surgical findings. A comprehensive review of the literature on medical/surgical management of patients with this rare finding will be presented.

## Case Report

A 32-year-old nulligravida Hispanic female was referred to our department with complaints of general malaise, abdominal distention, loss of appetite, diffuse abdominal pain and difficulty breathing that had worsened over the last few days. She was known to have endometriosis that was diagnosed at the time of an exploratory laparotomy due to massive haemorrhagic ascites performed two years before. She was started on oral contraceptives at that time with poor response and was subsequently treated with monthly 3.75 mg leuprolide IM (Lupron®) but she selfdiscontinued the treatment due to the desire to conceive.

The patient provided consent for publication of the case report. The IRB was consulted and the IRB committee at Hospital Pedro Mallo, Buenos Aires, Argentina deemed this work exempt of approval.

Initial imaging with ultrasound and computed tomography (CT) scan revealed a large amount of intraperitoneal fluid. A paracentesis was performed that obtained 5 litres of thick bloody peritoneal fluid with a red blood cell count of >50000/µL that was negative for bacteria or malignant cells. The patient had symptomatic relief and was discharged home after the procedure. She then returned eight days later complaining of recurrence of the same symptoms. A repeat ultrasound was performed along with magnetic resonance imaging (MRI), which revealed massive ascites (Figes.1, 2). She was taken to the operating room for diagnostic laparoscopy and drainage of the hemoperitoneum. Upon entry of the peritoneal cavity, a large amount of bloody peritoneal fluid was identified. We removed ten litres of hemoperitoneum (Fig .3). Extensive pelvic adhesions with complete obliteration of surgical planes was noted (Fig .4). The pelvis was described as “frozen” due to encapsulating peritonitis that prevented the creation of surgical planes (Fig .5). Multiple peritoneal biopsies were taken which revealed endometriotic implants (Fig .6). The patient had an uneventful postoperative recovery and was treated with the gonadotropin-releasing hormone (GnRH) agonist triptorelin (3.75 mg intramuscular injection prior to discharge. At three months of the postoperative course, the patient was asymptomatic without recurrence of the disease.

**Fig 1:**
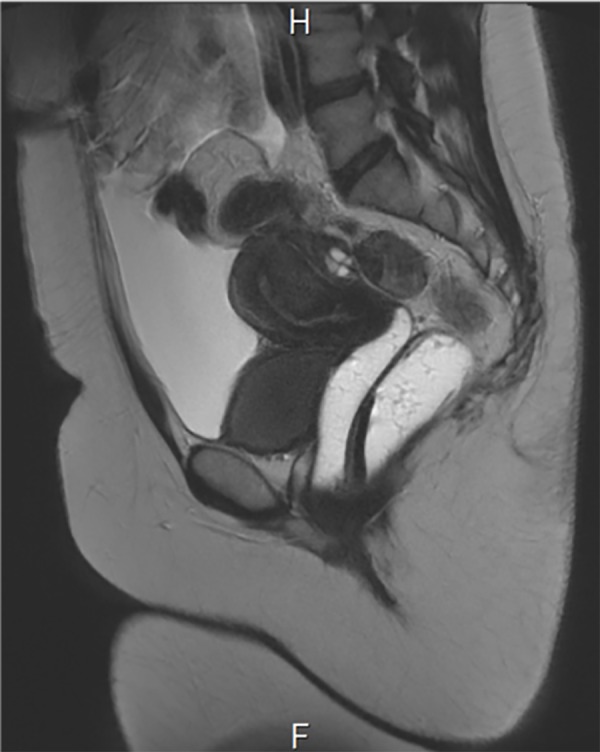
A large amount of intraperitoneal fluid is visualized on computed tomography (CT) of the abdomen and pelvis.

**Fig 2:**
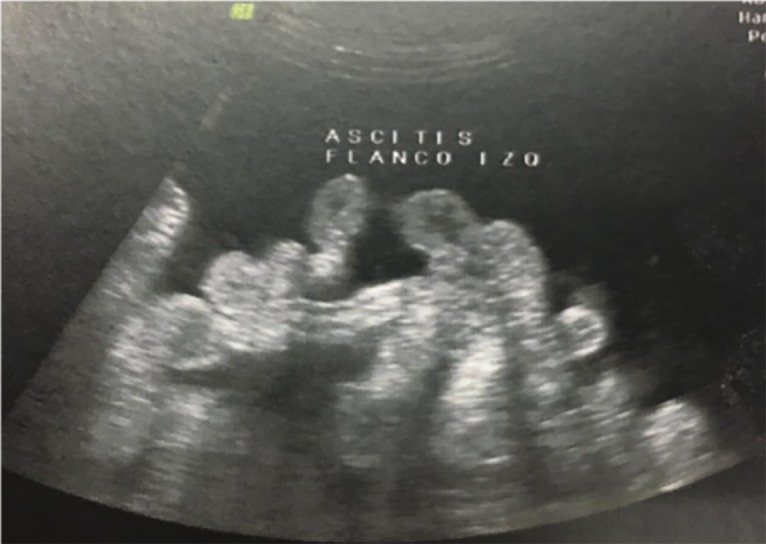
Massive ascites with small intestine floating inside the peritoneal cavity visualized on transabdominal ultrasound.

**Fig 3:**
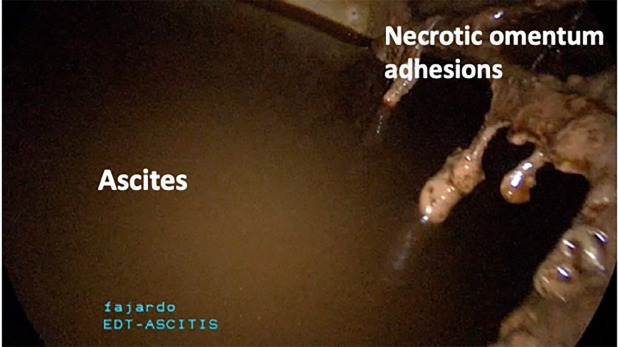
Bloody ascites filling the abdominopelvic cavity. Note necrotic omental adhesions on the anterior abdominal wall.

**Fig 4:**
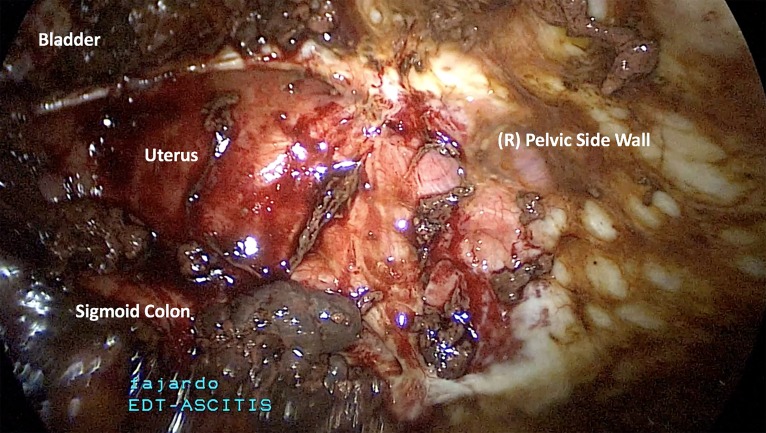
Note the complete obliteration of the vesicouterine space. The uterus is encapsulated from dense inflammatory plastic peritonitis and densely adheres to the pelvic side walls.

**Fig 5:**
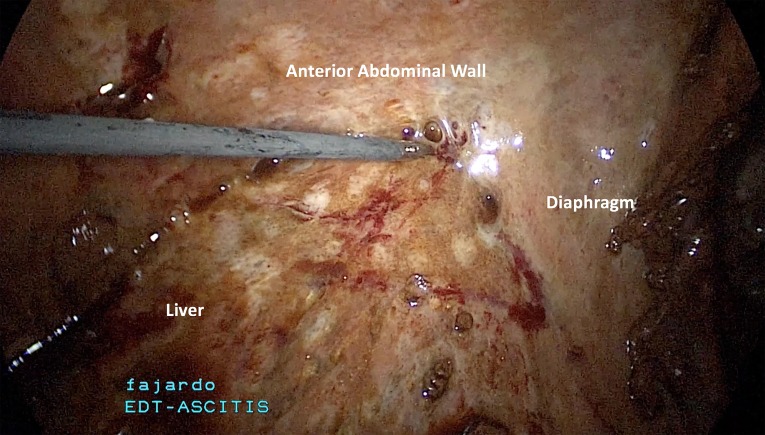
The liver is encapsulated by a dense parietal peritoneal inflammation. The liver is densely adherent to the anterior abdominal wall and the gallbladder is not visualized.

**Fig 6:**
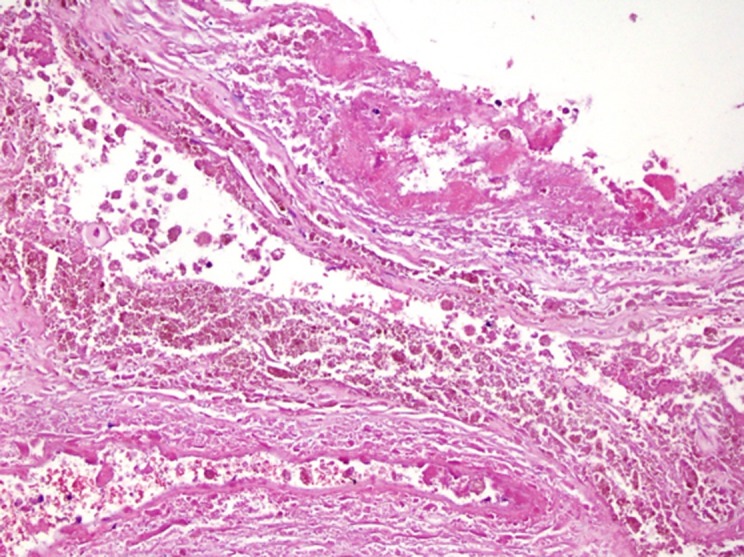
Peritoneal biopsy confirming the diagnosis of endometriosis. The endometriosis glands with periglandular endometriotic stroma that contain blood vessels are visualized.

## Discussion

Massive ascites associated with endometriosis is extremely rare with less than 100 cases described in the literature (9). Endometriosis is a common challenging condition of reproductive-age women. The spectrum of the disease ranges from asymptomatic to complete debilitation, which requires both aggressive surgical and medical intervention. As stated above, the most common presenting symptoms of endometriosis are dysmenorrhea and pelvic pain. Our case of haemorrhagic ascites represents an incredibly rare complication associated with endometriosis. Patients with haemorrhagic ascites typically present with weeks to months of increasing abdominal pain, anorexia/weight loss, abdominal pain and dysmenorrhea. This presentation often leads to a workup for malignancy as ovarian cancer was the suspected diagnosis in more than half of the patients who presented with haemorrhagic ascites (8).

While the majority of patients with haemorrhagic ascites present with a gradual onset of symptoms, reports of acute onset of symptoms have been published. A 2013 case report described a 27-year-old who presented with a one day onset of neck and flank pain, abdominal distention, light-headedness and palpitations. She was initially stable, but progressively decompensated and required transfusion of numerous units of packed red blood cells. Ultimately, a diagnostic paracentesis was performed and 4.5 litres of grossly bloody ascitic fluid was removed (10). Our patient who presented with an acute recurrence following drainage via paracentesis provided evidence of how quickly the hemoperitoneum can accumulate.

Patients with haemorrhagic ascites often pose a difficult diagnostic dilemma on initial presentation. The different diagnosis must include large haemorrhagic ovarian cyst rupture, ovarian cancer, ectopic pregnancy, endometriosis, Meigs’ syndrome, trauma, or other processes that could cause large hemoperitoneum. If necessary, initial stabilization measures with IV fluids and possible transfusion of blood products should be performed. As this presentation is so rare, no agreed upon workup is in place, but should be focused on ruling out the more common causes of hemoperitoneum. In a review of the literature, laboratory analyses that include complete blood count (CBC), basic metabolic panel (BMP), urine pregnancy and Ca- 125 were typically performed, along with basic imaging with either ultrasound, CT scan or MRI (8). Choice of imaging is often physician dependent; however, MRI is being used more frequently in evaluation for patients with this presentation (11).

Haemorrhagic ascites has been treated both medically and surgically. Medical management was attempted in 97% of patients with the use of hormonal therapy (e.g., GnRH agonist, danazol, progesterone, combination oral contraceptive pills or a combination of these) (8). These medications aim to inhibit ovarian functions and have been well documented to successfully treat endometriosis. Although medical management was attempted, 89% of patients ultimately underwent a surgical procedure (8). The average volume of ascites was 4470 ± 2625 mL (12). A review of numerous case reports showed that patients underwent a variety of surgical procedures, which varied from exploratory laparotomy with excision of an adnexal mass, total abdominal hysterectomy, oophorectomy, ovarian wedge biopsy, lysis of adhesions, or a combination of these. Newer case reports have also been published that show successful management via a laparoscopic approach, and one via diagnostic and therapeutic paracentesis (9, 10, 13). Improvements were seen with both medical and surgical management; however, as in our patient, recurrence is possible. The most successful treatments were bilateral salpingo-oophorectomy or ovarian suppression therapy. Both treatments had no recurrence of ascites (12).

The exact cause of haemorrhagic ascites in patients with endometriosis is unknown. It has been suggested that the ascites is caused by a ruptured endometrioma or by exudation of widespread pelvic endometriosis. However, Ussia et al. (12) reported that endometriomas were only seen in 65% of cases, and that widespread superficial pelvic endometriosis was only associated with a minimal increase in peritoneal fluid and not with massive ascites. They have stated that the pathophysiology is ovarian in nature and due to excessive ovarian transudation (e.g., similar to Meigs’ syndrome and Pseudo-Meigs’ syndrome). Meigs’ syndrome is based on the triad of an ovarian fibroma, pleural effusion and ascites with resolution of symptoms after resection of the fibroma. Pseudo-Meigs’ syndrome is associated with a benign pelvic mass and a typical rightsided pleural effusion without ascites (14, 15). Their case is strengthened by a 50% recurrence rate in the setting of unilateral oophorectomy or cystectomy compared to no recurrences when a bilateral oophorectomy was performed. Patients placed on ovarian suppression therapy with a GnRH agonist also had no recurrence during the time they were taking the medication.

Management needs to take into account a patient’s age, surgical history, medical history and future fertility plans. In patients who have no desire for future fertility and desire definitive surgical treatment, a bilateral salpingo-oophorectomy would be most effective. Subtotal surgical management (e.g., unilateral oophorectomy or cystectomy) alone should be avoided as the recurrence rate is high. Medical management with GnRH agonists are proven to be highly effective and should be used with a patient who desires future fertility, and for those who want to avoid surgical intervention.

## Conclusion

Haemorrhagic ascites is a poorly understood and rare manifestation of pelvic endometriosis. The differential diagnosis includes a variety of benign conditions, but malignant pathology must be ruled out. There are no specific protocols for the treatment of this rare condition. Current theories regarding the pathophysiology point to the ovary and excessive ovarian transudation. Management therefore involves surgical removal of bilateral ovaries or medical management with ovarian suppression. Patients who desire future fertility should be managed with a GnRH agonist. Clinicians should consider endometriosis in the differential diagnosis on female patients of reproductive age who present with haemorrhagic massive ascites.
